# Thermo-mechanical characterization of shale using nanoindentation

**DOI:** 10.1038/s41598-021-98251-x

**Published:** 2021-09-22

**Authors:** Yanbo Wang, Debora Lyn Porter, Steven E. Naleway, Pania Newell

**Affiliations:** 1 Integrated Multi-Physics Lab, Department of Mechanical Engineering, The University of Utah, Salt Lake City, 84102 USA; 2 Bioinspired Science and Engineering, Department of Mechanical Engineering, the University of Utah, Salt Lake City, 84102 USA

**Keywords:** Mechanical engineering, Materials science

## Abstract

Shale can be a potential buffer for high-level radioactive nuclear wastes. To be an effective buffer while subject to waste heat, shale's mechanical response at elevated temperature must be known. Many researchers have experimentally characterized the mechanical behavior of various shales at different length scales in adiabatic conditions. However, its mechanical performance at elevated temperatures at the nano-scale remains unknown. To investigate the temperature dependency of nanomechanical properties of shale, we conducted both experimental and numerical studies. In this study, we measured mechanical and fracture properties of shale, such as hardness, elastic modulus, anisotropy, and fracture toughness from 25 °C up to 300 °C at different bedding planes. Statistical analysis of the results suggests that hardness and fracture toughness significantly increased at temperatures from 100 to 300 °C; while, temperature does not have a significant impact on elastic modulus. Data also shows that the bedding plane orientations have a substantial impact on both mechanical and fracture properties of shale at the nano-scale leading to distinct anisotropic behavior at elevated temperature below 100 °C. Additionally, we numerically investigated the mechanical performance of the shale samples at room temperature to gain an insight into its mechanical response through the thickness. Numerical results were validated against the experimental results, confirming the simulation can be used to predict shale deformation at the nano-scale or potentially be used in multi-scale simulations.

## Introduction

There is a general agreement that deep geological disposal is currently one of the most appropriate solutions for the long-term management of high-level radioactive wastes^1^. The purpose of geological disposal is to isolate nuclear wastes from the biosphere and to ensure that the risk of human intrusion is low^2^. To contain radioactive waste safely, there are several desired properties for the buffer of the radioactive wastes: a low hydraulic permeability, a self-sealing ability, and durability of properties^3^. One such buffer is shale, which contains silicates, carbonates, and clay minerals. The clay minerals in shale can seal nuclear waste underground because of their low hydraulic permeability. When the clay particles in the shale come in contact with moisture and swell, the shale self-seals and becomes a better container for the nuclear waste^3,4^.

To safely store nuclear waste, it is important to understand the mechanical properties of shale formations. However, due to the presence of features at different length scales, an in-depth understanding of shale’s performance as a buffer for nuclear waste requires a comprehensive knowledge of its performance across scales. Traditional geomechanics testing such as the uniaxial compression test, triaxial test, etc. can be used to measure macroscopic mechanical properties of shale. For instance, Islam et al. conducted a triaxial test at room temperature and showed that the elastic modulus of Pierre-1 shale varies with respect to the orientation angles^5^. Lora et al. conducted isotropic compression tests of Marcellus Shale and highlighted its anisotropic nature as well as the dependency of its mechanical response on the pressure magnitude^6^. Gao et al. experimentally characterized the geomechanical properties of shales from the Ohio region at the macro-scale and illustrated that the compressive strength of their samples was much higher than their tensile strengths^7^. Natural fractures play a significant role in rock formations, and they influence their mechanical and fracture properties. For instance, Yang et al. studied nature fractures, and mechanical properties of Horn River Shale at the macro-scale based on calculating the geometries of fractures^8^. They illustrated hardness values are more reliable to predict the distribution of natural fractures^8^.

On the other hand, nano-scale mechanical properties of materials can be measured through non-traditional methods such as nanoindentation, which is an experimental method for testing the surface mechanical properties at the nanometer scale with high-precision instrumentation^9–11^. Shi and Liu used the nanoindentation method to experimentally characterize the micro-scale mechanical behavior of various shales at room temperature^12–14^. Shi et al. demonstrated that mechanical parameters perpendicular to the bedding plane are slightly lower than those parallel to the bedding plane^12^. They also tested mechanical properties of Longmaxi formation shale samples before and after exposure to supercritical $\hbox {CO}_{2}$ and found out that the mechanical properties (i.e., hardness, elastic modulus, and fracture toughness) decreased after the treatment^13^. Liu et al. calculated fracture toughness of Bakken formation shale at the micro-scale and observed that fracture toughness increases linearly with elastic modulus^14^.

Due to the radioactivity of nuclear waste, underground nuclear waste storage will heat the surrounding shale rocks, which will likely result in associated changes in their mechanical properties. Therefore, some researchers have focused on the role of temperature on shale’s mechanical properties. Sharma et al. studied the effect of temperature on the creep properties of organic-rich shales at both nano and micro-scales and found creep properties are affected by the removal of clay-bound water above 200$^{\circ }$C^15^. Other researchers studied macro-scale fracture behavior of shales at higher temperatures up to 500 °C and concluded that the temperature change tends to substantially alter the fracture behavior as microcracks are formed within the shale rock^16,17^. Others showed that the elastic modulus decreases significantly with an increase in the temperatures.

Masri and Chandler studied temperature dependency of Tournemire and Mancos shale mechanical behavior at the macro-scale and observed a considerable decrease of elastic modulus and a slight increase of fracture toughness with increasing temperature^18,19^.

Despite many experimental and numerical investigations of mechanical properties of various shale types for different geological applications, there still exists a lack of knowledge in understanding its nano-mechanical and nano-fracture properties at elevated temperatures, which is essential in understanding the long-term performance of any nuclear waste repositories. Therefore, this study will exclusively look at these properties with respect to bedding plane and temperature variation.

Researchers have shown that the free water in shale is accessible at 100$^{\circ }$C and the interlayer water can be reached at temperatures around 200$^{\circ }$C, thus we selected a temperature range from room temperature to 300$^{\circ }$C^20–22^.

## Results and discussion

### Mineral compositions and microstructures

**Figure 1:**
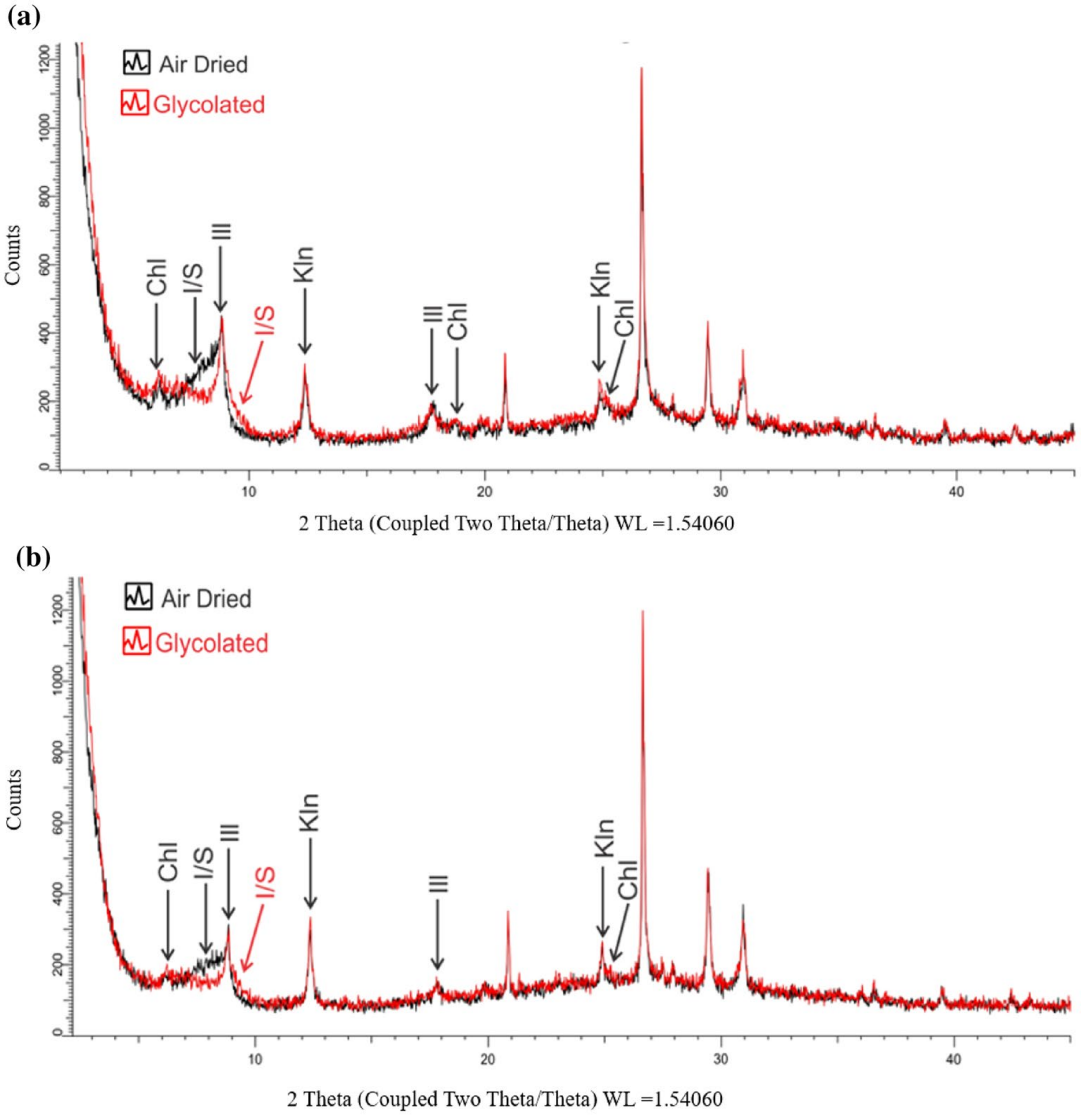
(**a**) XRD analysis of clay-sized fraction for both air dried and glycolated treatments in vertical pattern. (**b**) XRD analysis of clay-sized fraction for both air dried and glycolated treatments in horizontal pattern. (Glycolated: treated with ethylene glycol) [Illite (Ill), illite/smectite (I/S), Kaolinite (Kln), Chlorite (Chl)].

Shales are known to be heterogeneous in nature due to the presence of various mineral compositions. Thus, to understand its overall mechanical properties, it is important to know its mineral components. X-Ray Diffraction (XRD) analysis, a common technique that reveals the chemical composition of a material, was performed on our samples. Figure 1 shows this comparison for both vertical and horizontal bedding planes indicating that they contain the same amount of clay minerals. The mineralogy of the clay fraction is then used in the Rietveld refinement of the bulk sample to quantify the abundance of the crystalline phases. Table 1 gives the mass percentage of the sample, with results rounded to the nearest whole number. Fields marked with tr (trace) indicate that mineral is present in the clay-sized fraction (Fig. 1), but not detectable in the bulk XRD patterns and/or its calculated value was less than one weight percent. The results highlight that, in both horizontal and vertical bedding planes, the rock is mainly composed of quartz minerals (quartz, k-feldspar, plagioclase), carbonate minerals (calcite, dolomite), and clay minerals (illite, kaolinite, chlorite), but also contains a small amount of pyrite.

**Table 1:** XRD analysis of the mass fraction of mineral components in shale samples for vertical and horizontal bedding planes.

Sample	Silicates			Carbonate		Clay minerals				Pyrite
	Quartz	K-feldspar	Plagioclase	Calcite	Dolomite	Illite	Interlayered illite/smectite	Kaolinite	Chlorite	
Vertical pattern (mass fraction/%)	46	6	5	7	11	9	7	8	tr	tr
Horizontal pattern (mass fraction/%)	57	6	4	7	10	6	4	6	tr	tr

**Figure 2:**
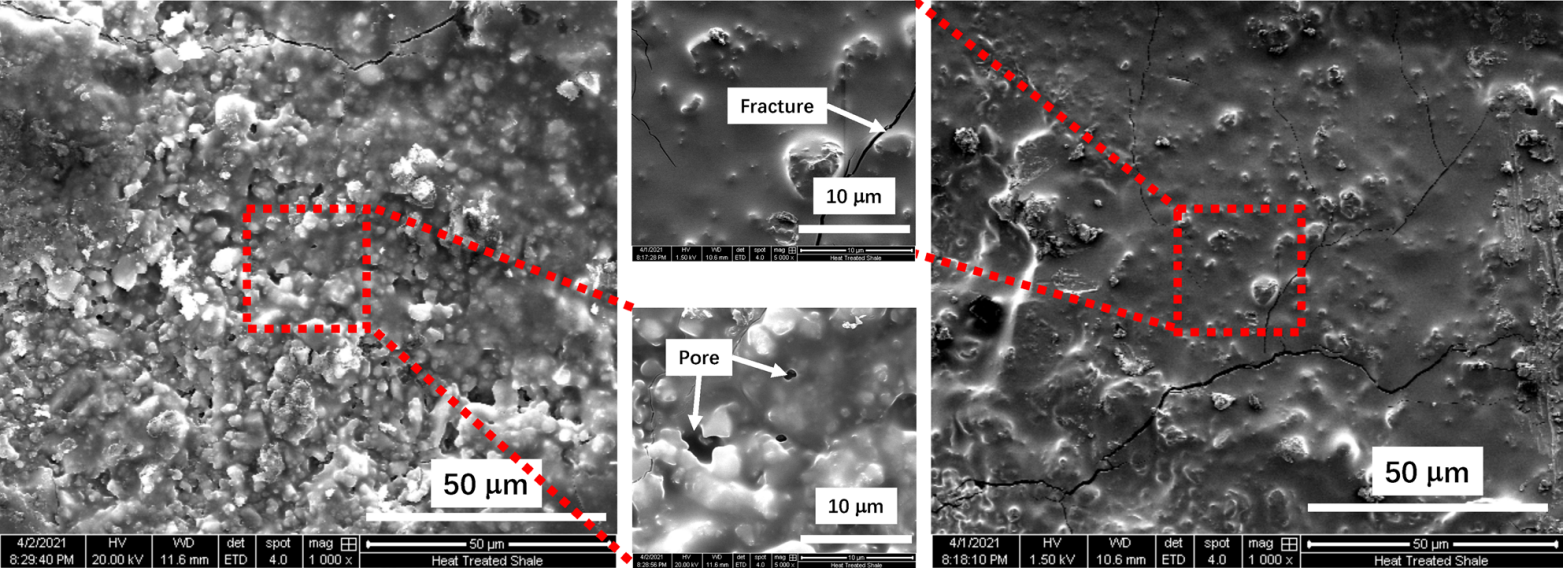
SEM images of the pores and fractures within the shale sample after thermal exposure.

Figure 2 shows the pores and microfractures on the surface of our shale samples after thermal exposure. These microcracks are mainly because of temperature change and temperature gradient in the shale causing thermal stress. Thermal cracking was also reported by Kang^20^, where they heated a shale sample from the Longmaxi formation to 800 °C and found that the sample’s mass decreased before 300 °C but it remained unchanged after 300 °C. They indicated that this mass decrease was due to the loss of the absorbed and interlayer water in the shale. Furthermore, they explained that the instant evaporation of interlayer water resulted in mineral crystal fracture and made the material less compliant, which would in turn result in the creation of micro/nano fractures^20^.

### Elastic modulus and hardness

To understand the role of temperature on the mechanical properties of our samples, we ran a total of 200 tests at different temperatures (i.e., 25 °C, 75 °C, 100 °C, 300 °C) and we measured hardness, elastic modulus in both vertical and horizontal bedding planes. We performed nanoindentation at multiple temperatures by utilizing a state-of-the-art thermal stage within the nanoindenter, which enables quantitative, accurate, and reliable nanomechanical characterization at elevated temperatures up to 800 $^{\circ }$C.

**Figure 3:**
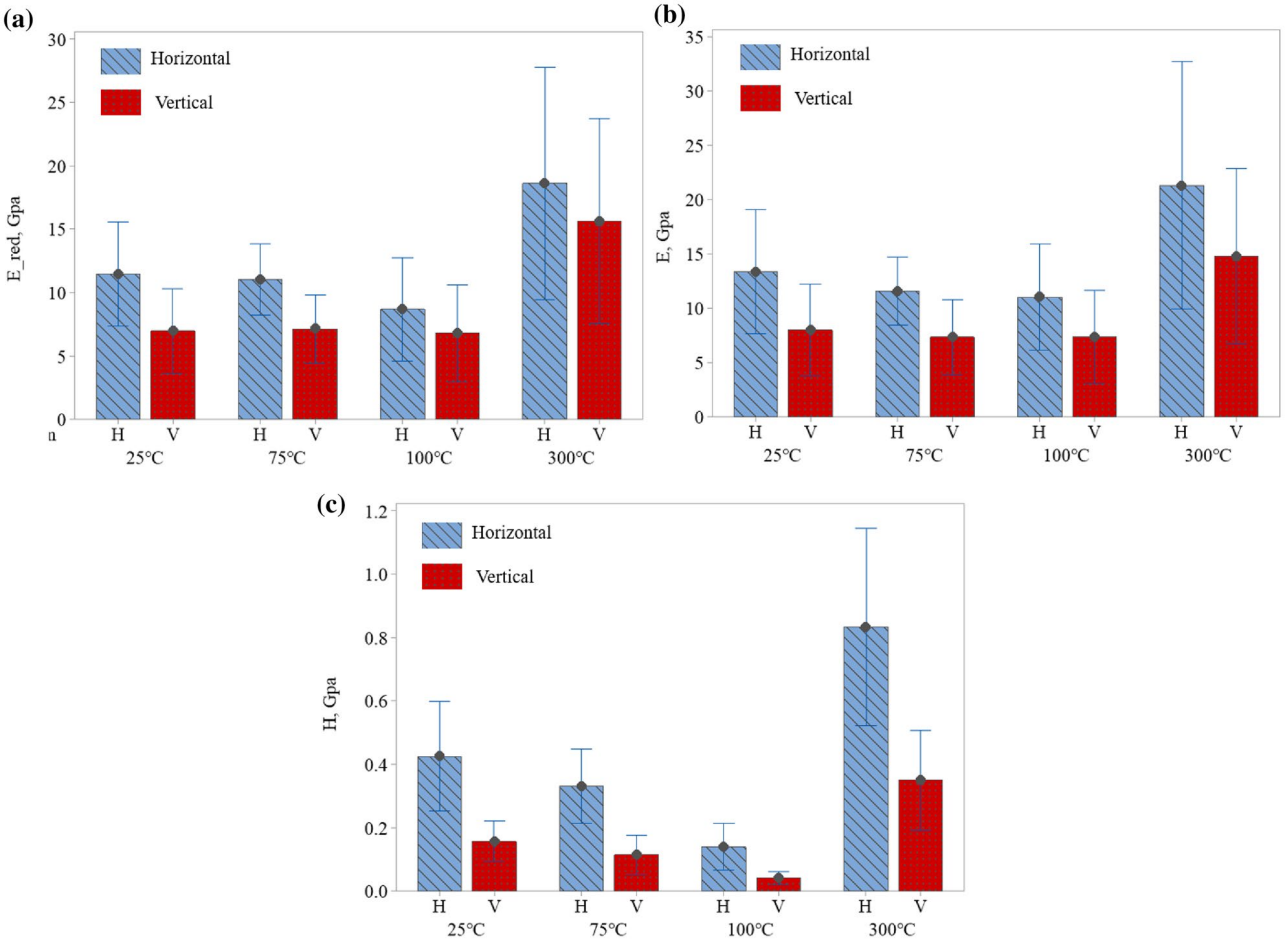
(**a**) Reduced modulus for both vertical and horizontal bedding planes in elevated temperatures. (**b**) Elastic modulus for both vertical and horizontal bedding planes in elevated temperatures. (**c**) Hardness values for both vertical and horizontal bedding planes in elevated temperatures.

Figure 3a–c show the reduced modulus, elastic modulus, and hardness values, respectively, at different temperatures for samples cut in vertical and horizontal bedding planes. The reduced modulus (Fig. 3a) and elastic modulus (Fig. 3b) are related by Eq. (6). These figures show that the elastic modulus and hardness values in the horizontal bedding plane are always higher than their corresponding values in the vertical bedding plane for all of our selected temperatures. This observation is also confirmed by statistical analysis (p < 0.001), indicating a significant impact of the orientation of the bedding plane on both elastic and hardness values. Moreover, our results at room temperature are in agreement with the results reported by others^12,23^. Rybacki et al. studied the macro-scale mechanical properties of European black shale with the uniaxial compression method. They found that the elastic modulus and strength in the vertical bedding plane are higher than the horizontal bedding plane, which is the same trend shown in our nano-scale study^24^. Such orientation dependency is the same across length scales and can be explained based on the compaction deformation of bedding planes, which makes the mechanical properties in the horizontal bedding plane higher than the vertical one^18^. At higher temperatures, the dehydration strengthens the shale rock and increases the elastic modulus. These characteristics of the shale shed light on the ability of shale rocks to store high-temperature nuclear waste.

In statistics, p-value is the probability of finding the results when the null hypothesis is true. The lower p-value means the greater statistical significance of the observed difference. The statistical analysis also indicates that the hardness of our shale samples is influenced by temperature (p < 0.001). More detailed analysis (i.e., Tukey’s HSD test) reveals that hardness value does not show a significant dependency on temperatures below 75 °C. On the other hand, the statistical analysis suggests that the elastic modulus of our samples is not significantly affected by the temperature (p $=$ 0.625) within our selected temperature range. It should be noted that we assumed Poisson’s ratio remained constant at different temperatures because the reduced modulus and elastic modulus in Fig. 3 have a similar trend. Both the reduced modulus (p $=$ 0.473) and the elastic modulus (p $=$ 0.625) are not significantly affected by temperature.

**Figure 4:**
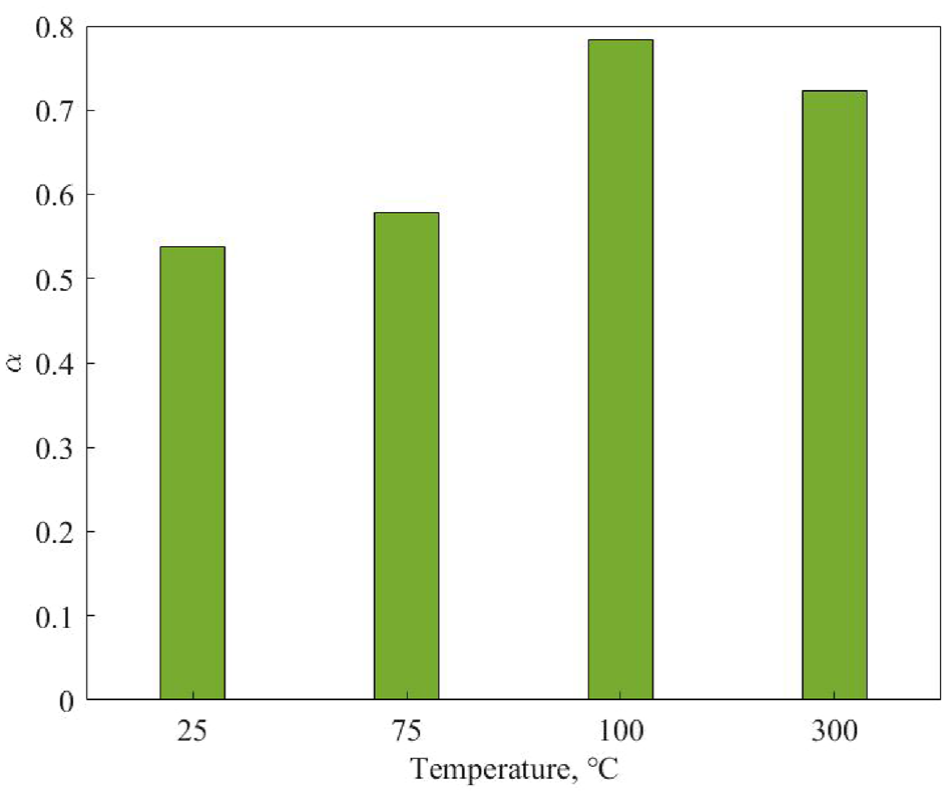
Variation of anisotropic coefficient at elevated temperatures.

Moreover, we define the anisotropic coefficient as

1 $$\begin{aligned} \alpha =\frac{E_{v}}{E_{h}} \end{aligned}$$

where $E_{v}$ and $E_{h}$ are the elastic modulus in vertical and horizontal bedding planes, respectively. If we use the average values of each bedding plane at different temperatures, one can plot the variation of anisotropic coefficient in terms of temperature as shown in Fig. 4. This figure shows at temperatures below 100 °C; there is higher anisotropy in our samples, which picks at 100 °C where most of the accessible water is evaporated. This value drops down at 300 °C, which is an indication that interlayer pore spaces are more accessible in the vertical cases than the horizontal ones. This knowledge is important as the anisotropic characteristic of shales influences the thermal field as well as cracking pattern and should be considered in long-term analysis of nuclear waste repositories as well as other geological applications where shale formations are involved.

### Fracture toughness

**Figure 5:**
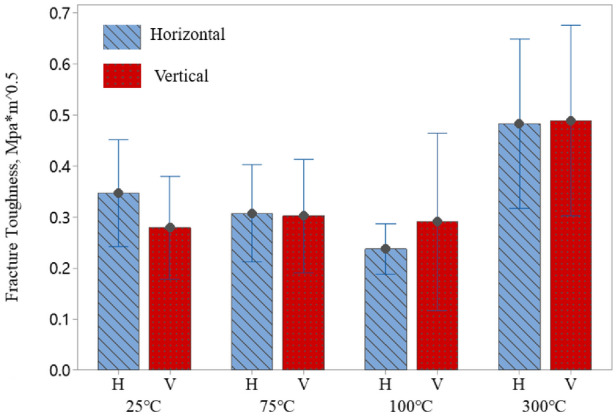
Fracture toughness values of shale samples in both vertical and horizontal bedding planes.

Figure 5 shows fracture toughness values calculated based on the energy analysis method, where 25 indentation tests were conducted at four different temperatures (i.e., 25 °C, 75 °C, 100 °C, and 300 °C). Statistical analysis of the obtained results show that the fracture toughness depends on the orientation of the bedding plane (p $=$ 0.046), which is due to the variation in water content in the horizontal and vertical orientations. Furthermore, a closer look along with more detailed analysis (i.e., Tukey’s HSD test) reveals that the fracture toughness is only influenced when the temperature changes from 100 to 300 °C. Since our shale samples with different bedding planes are from the same area of the San Juan Basin and the mineral components are the same according to the XRD analysis (Table 1), the difference in fracture toughness values at temperatures from 100 to 300 °C is likely due to the water removal from the samples.

It should be noted that Chen et al. studied the effect of water on fracture properties of shales at room temperature, and found a distinct reduction in fracture toughness with an increase in the water content of shale^25^. Chandler et al. studied the effect of temperature on the fracture toughness of shale rocks using the short-rod methodology at the macro-scale and concluded that the fracture toughness changes very little until 120 °C, followed by a modest increase at temperatures between 120 and 200 °C^19^. This is an indication that dehydration of the samples influences the variation of fracture toughness at elevated temperatures leading to similar trends at the macro and nano-scales. We also conclude that although the fracture toughness of our shale samples at the nano-scale changed from 100 °C to 300 °C, there is no significant change from room temperature to 300 °C. This indicates that our shale samples can store the high-temperature nuclear wastes without much crack growth.

### Finite element analysis

**Figure 6:**
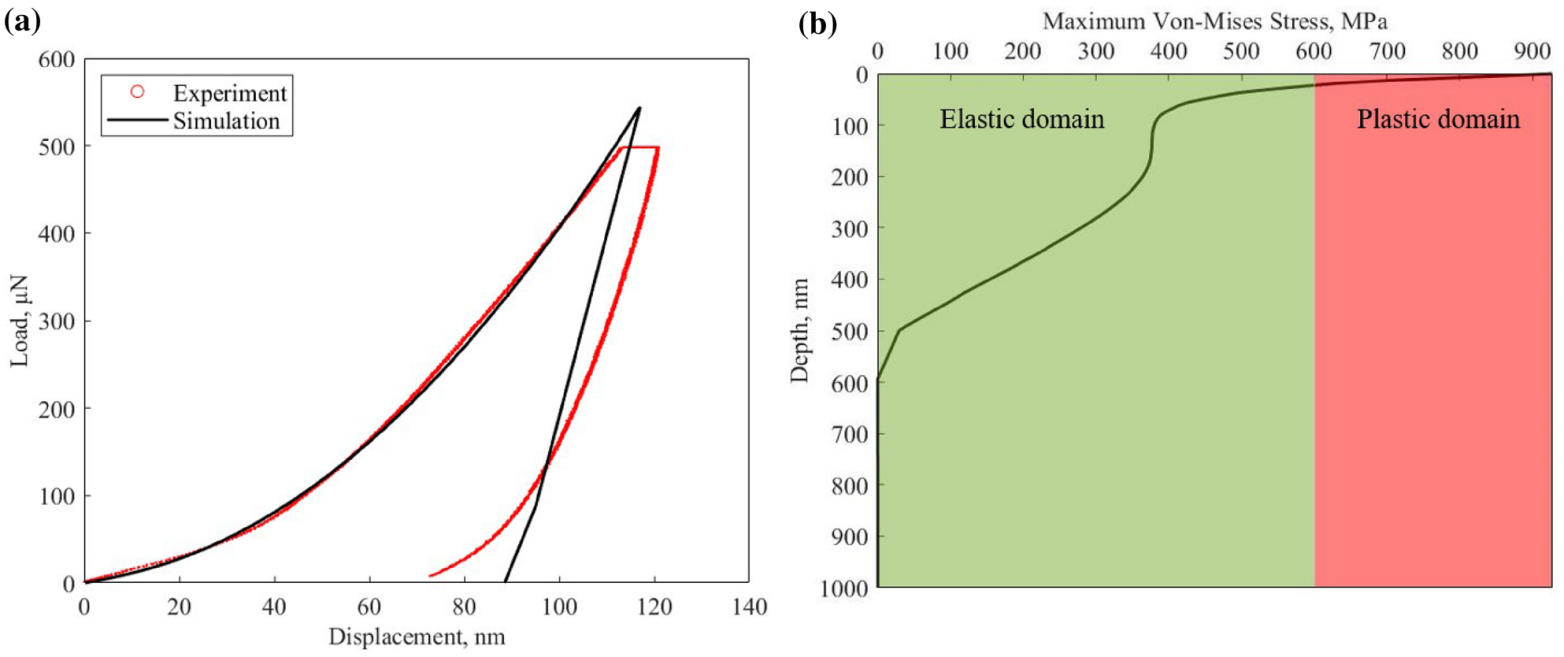
(**a**) Comparison of experimental and numerical load and displacement curve. (**b**) The Von-Mises stress changes depending on the indentation depth during the numerical study.

The finite element analysis can provide more information compared with the surface data obtained from the nanoindentation test. This information enables us to see what happens inside a sample during the indentation experiment. We modeled our sample’s deformation behavior under indentation at room temperature with a two-dimensional axisymmetric model using ANSYS software^26^ (ANSYS-Academic V19.2, https://www.ansys.com/academic/students). The elastic modulus and Poisson’s ratio were used as the input of the simulation. Fig. 6a shows a good agreement between the simulation and experimental results. The maximum load in simulation is around 544.23 μN, which is higher than the experiment’s 500 μN maximum load. This is mainly due to the fact that during the holding section region of the experiment (described in the “Nano-indentation method” section), the indenter will continue to propagate inside the sample; however, our numerical model cannot characterize the creep behavior. The creep behavior in the experiment makes the maximum displacement different in the numerical simulations. To have the same maximum displacement as the experiment, the numerical model must have a higher load than the experiment. Similar numerical results have also been observed in the literature^27^. The loading curve matches accurately when the input yield stress and tangent modulus are 600 MPa and 550 MPa, respectively. The initial slope of the unloading curve in the nanoindentation experiment represents the stiffness of the material. The difference between the shape of the unloading curves is due to our choice of linear elastic constitutive model that cannot capture nonlinear behavior. To fully characterize the nanoindentation experiment, a more robust constitute model and time-dependent plasticity should be incorporated into the simulation.

Von-Mises stress is a value used to determine whether a given material will yield, and the input yield stress of the shale sample in our simulation is 600 MPa. Figure 6b shows how maximum Von-Mises stress varies beneath the indentation point along the y-axis throughout the simulation (see Fig. 10). In this figure, the green and red regions are elastic and plastic domains, respectively. As one can see, at depth of $\sim$ 25 nm below the surface, materials experience plastic deformation, which indicates that the majority of the microcracking associated with indentation will occur within this region. Furthermore, the Von-Mises stress below 600 nm seems to be negligible.

## Closing remarks

In this study, for the first time, we conducted a coupled thermo-mechanical analysis of Mancos shale at the nano-indentor. The XRD results (Table 1) showed our samples had similar compositions in different bedding orientations. Since the mineral components are the same in both horizontal and vertical bedding planes, the performance of the shale in different bedding orientations will be similar after being exposed to different temperatures. The SEM images (Fig. 2) show examples of microcracks that were formed in our samples due to temperature changes. The statistical analysis of the data revealed that the bedding plane has a pronounced impact on the elastic modulus, hardness, and fracture toughness independent from the temperature. Furthermore, only hardness values were significantly influenced by temperatures above 75$^{\circ }$C, while elastic modulus and fracture toughness were slightly impacted by the change in the temperature. There is also a pronounced anisotropy in our samples below 300$^{\circ }$C. Overall, the influence of temperature on mechanical and fracture properties is scale-independent and follows the same trend in both nano and macro-scales. Although, nano and macro-scale have similar trend, the mechanical properties at different scales are different. Auvray et al. studied the elastic modulus of claystone across scales and demonstrated the elastic modulus of the claystone is scale depended^28^. Shi et al. compared the mechanical properties of Longmaxi formation shale from the uniaxial compression test and the nanoindentation test. They concluded that the nano-scale calculation is lower because of the micropores and fractures inside the shale samples^12^. Our numerical analysis also indicated that below 600 nm from the surface of the samples, the materials remain elastic at room temperature.

## Methods

### Nano-indentation method

**Figure 7:**
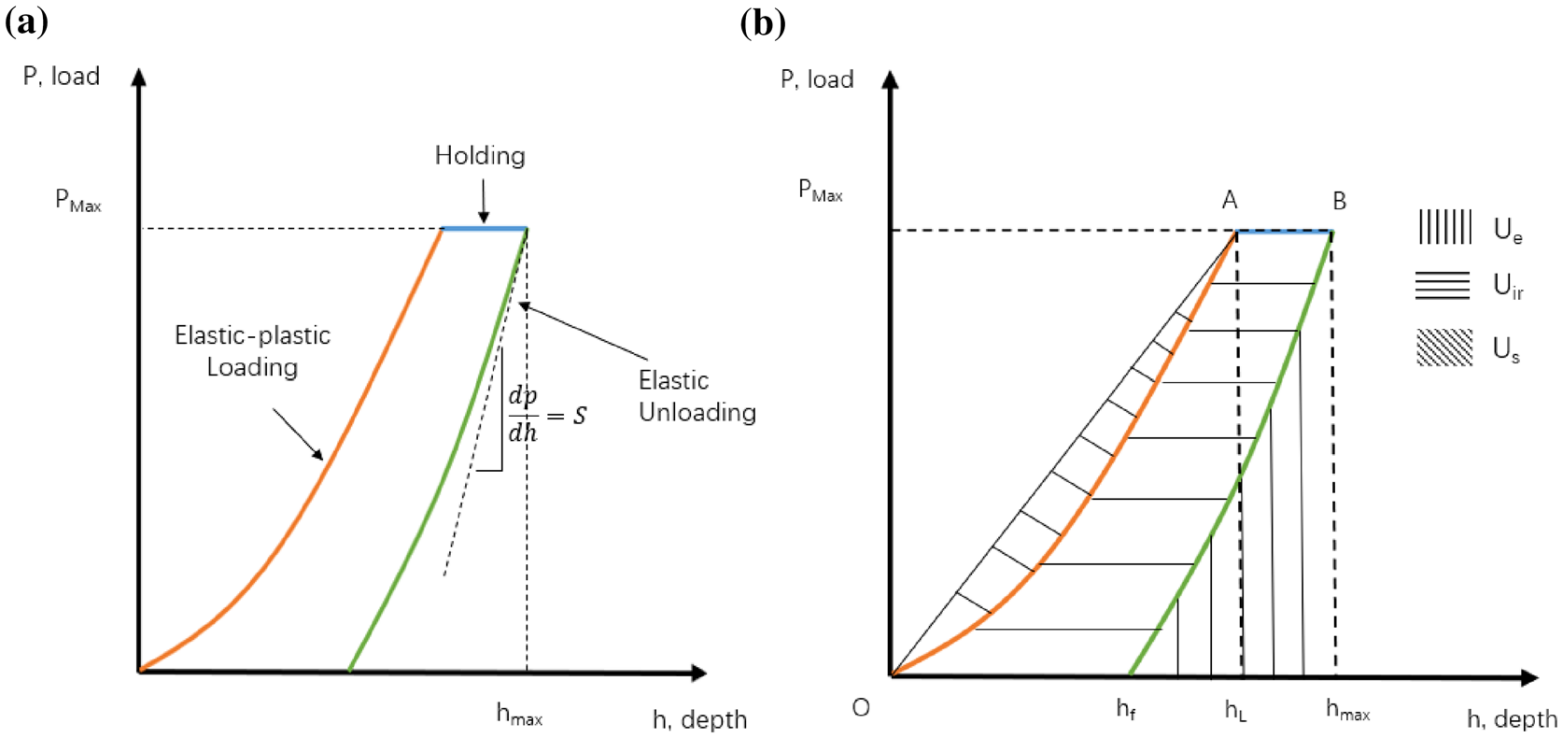
(**a**) Typical load-displacement curve obtained from nanoindentation test. This curve highlights three distinct regions of: loading, holding, and unloading. (**b**) Typical load-displacement curve obtained from nanoindentation highlighting different areas associated with elastic, irreversible, and absolute energy^14^.

Figure 7a shows a typical load-displacement curve obtained from a nano-indentation experiment. There are three regions in each nanoindentation test: loading, holding, and unloading. In the loading region, both elastic and plastic deformations take place. The elastic deformation happens when a low load is applied to the specimen, and the material goes back to its original configuration after the load is removed. In contrast, the plastic deformation is defined as when the specimen is no longer recovers to its original configuration upon removal of the load. It should be noted that in the unloading region, we assume only the elastic deformation will be fully recovered.

The stiffness is a structural property that measures how well an object resists elastic deformation from an applied force. Therefore, higher values of *S* indicate a better ability of a material to return to its original shape. In nanoindentation, stiffness (also called initial unloading stiffness) is calculated based on the slope of the upper portion of the unloading curve (Fig. 7a) as:

2 $$\begin{aligned} S=\frac{dP}{dh}, \end{aligned}$$

The contact area (projected area) A($h_c$) is a function of the contact depth $h_c$ (Eq. 8):

3 $$\begin{aligned} A(h_c)=\sqrt{3}\tan ^2(\alpha )=24.5h_c^2, \end{aligned}$$

where $\alpha$ is a apical angle of the Berkovich indenter equal to 65$^{\circ }$. Once the stiffness and the contact area were obtained, the reduced modulus, $E_r$, can be calculated as

4 $$\begin{aligned} E_r=\frac{\sqrt{\pi }S}{2\sqrt{A(h_c)}}, \end{aligned}$$

After rearranging Eq. (4), we have

5 $$\begin{aligned} A(h_c)=\frac{\pi }{4}\left[ \frac{S}{E_r}\right] ^2, \end{aligned}$$

The reduced modulus, $E_r$, is also related to the elastic modulus of a sample $E_{sample}$. In the nanoindentation method, this mechanical property measures the tensile stiffness of a solid material and can be calculated based on:

6 $$\begin{aligned} \frac{1}{E_r}=\frac{1-v_{sample}^2}{E_{sample}}+\frac{1-v_{indenter}^2}{E_{indenter}}. \end{aligned}$$

where $E_{indenter}$ and $v_{indenter}$ are the elastic modulus and Poisson’s ratio of the indentor. For a standard diamond indenter probe, $E_{indenter}$ is 1140 GPa and $v_{indenter}$ is 0.07.

Through our nanoindentation experiment, we can also calculate hardness, which is the resistance to localized plastic deformation. Because solid material displays different behavior under different forces, there are several kinds of hardness: scratch hardness^29^, indentation hardness^30^, and rebound hardness^31^. Each hardness can be calculated using a maximum force of the particular indentation test as follows:

7 $$\begin{aligned} H=\frac{P_{max}}{A(h_c)}, \end{aligned}$$

where *H* is hardness, $P_{max}$ is maximum force, and $A(h_c$) is contact area. The contact depth is calculated

8 $$\begin{aligned} h_c=h_{max}-0.75 \times \frac{P_{max}}{S}, \end{aligned}$$

where $h_c$ is contact depth, $h_{max}$ is maximum depth, $P_{max}$ is maximum force, and *S* is stiffness. The 0.75 value is used to account for edge effects, including the deflection of the surface at the contact perimeter. The maximum contact area $A_{max}$ can be calculated by:

9 $$\begin{aligned} A(h_{max})=24.5h_{max}^2. \end{aligned}$$

The nanoindentation experiment can also provide information on the fracture toughness of the material. Fracture toughness is a material property that indicates the critical stress at which a crack in a brittle solid becomes unstable and propagates. It can also describe the ability of a material containing a crack to resist fracture^32^. It is worth mentioning that the calculation of the rock fracture toughness is an energy-based method and was originally introduced by Cheng^33^. Fracture energy ($U_c$) is defined as the difference between irreversible energy ($U_{ir}$) and pure plastic energy ($U_{pp}$).

10 $$\begin{aligned} U_c= U_{ir}-U_{pp}=U_{t}-U_{e}-U_{pp}, \end{aligned}$$

where $U_{pp}$ is the energy due to the pure plasticity, $U_{ir}$ is the irreversible energy determined from the area $OABh_{f}$ (Fig. 7b), $U_{e}$ is the elastic energy determined from the area $h_fBh_{max}h_f$ (Fig. 7b), and $U_{t}$ is the total energy. Irreversible energy ($U_{ir}$) is defined as the difference between the total energy ($U_{t}$) and the elastic energy ($U_{e}$). $U_{s}$ in Fig. 7b is the absolute work from the nanoindentation experiment determined by the area $OAh_{L}O$. The energy due to the pure plasticity $U_{pp}$ can be calculated by:

11 $$\begin{aligned} \frac{U_{pp}}{U_t}= 1-\left[ \frac{1-3\left( \frac{h_f}{h_{max}}\right) ^2+2\left( \frac{h_f}{h_{max}}\right) ^3}{1-\left( \frac{h_f}{h_{max}}\right) ^2}\right] . \end{aligned}$$

The critical energy release rate is the rate of energy transformed as a material undergoes fracture. Mathematically, the critical energy release rate is a decrease in total potential energy per an increase in fracture surface area^34,35^. The critical energy release rate $G_c$ is given by:

12 $$\begin{aligned} G_c=\frac{\partial U_{c}}{\partial A} =\frac{U_{c}}{A_{max}}, \end{aligned}$$

where $A_{max}$ is the maximum crack area, which can be calculated from Eq. (9). Finally, the fracture stress intensity factor $K_{C}$ can be computed as:

13 $$\begin{aligned} K_{C}=\sqrt{G_c \times E_r}. \end{aligned}$$

When the fracture stress intensity factor $K_{C}$ increases to a critical value, the crack in the material begins to grow. This critical value is called fracture toughness $K_{C}$ or $K_{IC}$ that is an indication of the material’s ability to resist unstable crack propagation and it is considered as a material property^36^.

### Experiment

Before performing the nanoindentation test, the tip area function was calibrated by several indentations on a standard fused quartz sample. During this experiment, the sample is gradually loaded until it reaches the maximum load. The maximum load of each point is 500 μN. Then we hold the load for 2 s before unloading to get a more accurate result. To automatically collect a group of indentation points, the grid indentation method was selected. The grid indentation technique is a good method to characterize the mechanical properties of heterogeneous material, and it has been applied to various shale samples^37,38^. In this study, the domain region was 200 $\times$ 200 μm^2^. We collected a 5 $\times$ 5 array of indents for a total of 25 indentation points for each temperature (100 points for each sample). These points are distanced by 50 μm to avoid interference.

**Figure 8:**
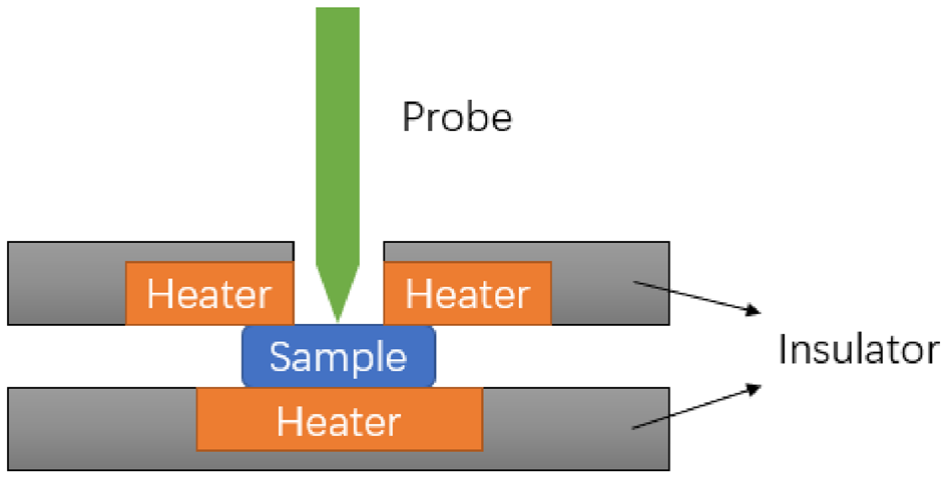
Schematic of the xSol Temperature Stage used in the TI 950 TriboIndenter systems which is capable of heating the sample from room temperature to 800 $^{\circ }$C.

The TI 950 TriboIndenter systems with the thermal stage shown in Fig. 8 were used to analyze the samples at elevated temperatures. The indenter is located in the Nanofab user facility at the University of Utah. The maximum able load is 100 mN. The maximum displacement and the maximum force for this experiment were set to 5 mN and 10 mN, respectively. The thermally stable xSol stage enables quantitative, accurate, and reliable nanomechanical characterization at elevated temperatures up to 800$^{\circ }$C.

**Figure 9:**
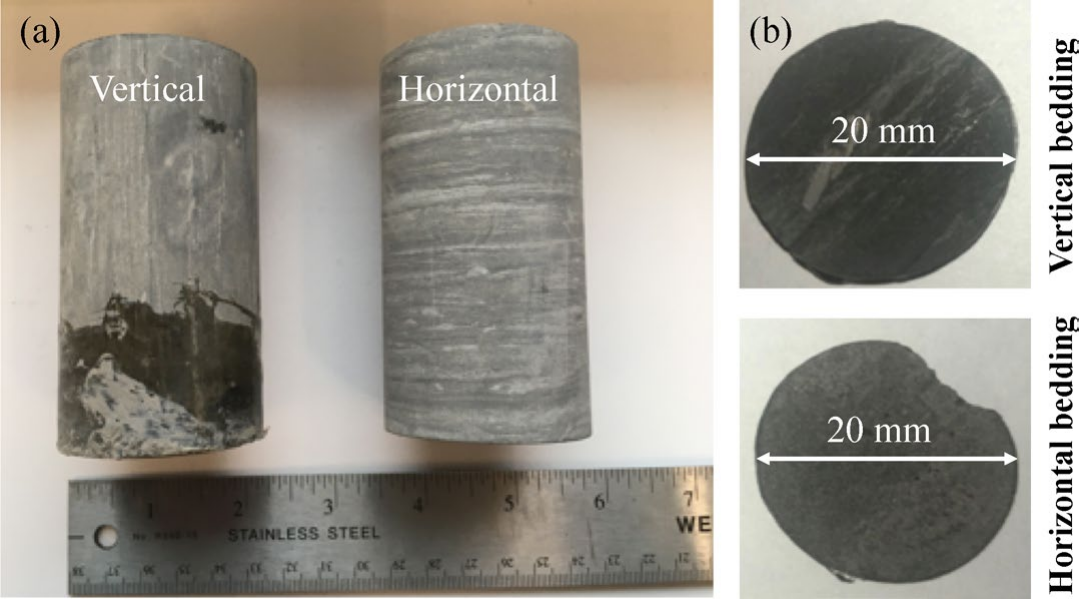
Polished samples cut from cores obtained along vertical and horizontal bedding planes.

The samples are from a thick section of Mancos shale from the San Juan Basin, located in southwestern Colorado and northwestern New Mexico. Specimens in this experiment are two shale samples cut along different orientation planes (i.e., vertical and horizontal) (Fig. 9a), and are all taken from the same large block of rocky outcrop. The samples were cut into small cylinders, 3-mm thick with a 20-mm diameter (Fig. 9b). Both sandpaper and diamond lapping film were used to polish the samples’ surfaces. The grit size on the paper dropped from 80 to 0.5 μm. To ensure uniform surface smoothness, we polished until a thickness three times the grit size was removed (Fig. 9b). The nanoindentation experiments were performed on the surface of the samples in Fig. 9b for the nano-scale mechanical properties.

To characterize the samples’ mineral components, the whole-rock and clay XRD analyses were performed on each sample using a Bruker D8 Advance X-ray diffractometer available at the Energy Geoscience Institute at the University of Utah. To identify the clay minerals present in the sample, we followed a method described by Moore, in which the air-dried, glycolated and heated scans of the clay-sized fraction are compared with each other^39^. Phase quantification using the Rietveld method was performed using TOPAS software^40^ (TOPAS-Academic V5.0, http://www.topas-academic.net/), developed by Bruker AXS. The Rietveld method fits the peak intensities calculated from a model of the crystalline structure to the observed X-ray powder pattern by a least-squares refinement^41^. This is done by varying the parameters of the crystal structures to minimize the difference between the observed and calculated powder patterns. Because the whole powder pattern is taken into consideration, problems of peak overlap were minimized and accurate quantitative analyses were obtained.

Quanta 600 FEI Scanning Electron Microscope (SEM) available at the Utah NanoFab user facility was used to obtain microstructure images of the samples. Samples were coated with 15 nm of gold-palladium and imaged using an acceleration voltage of 20 kV and a spot size of 4 nm.

### Simulation

We simulated an indentation model with a diamond shape probe, and a deformable flat surface is used to analyze the samples’ deformation performance under loading. In this method, the rigid tip is penetrated into the deformable plate by applying a load in the center of the tip. We used Ansys^26^ (ANSYS-Academic V19.2, https://www.ansys.com/academic/students), a commercial software package, to numerically analyze our nanoindentation test^42^. The indentation process is a quasi-static process. To simplify the model, the nanoindenter was modeled using a 2-D axisymmetric model. Using this model, compared to a 3D model, will considerably reduce the computation time^43^.

**Figure 10:**
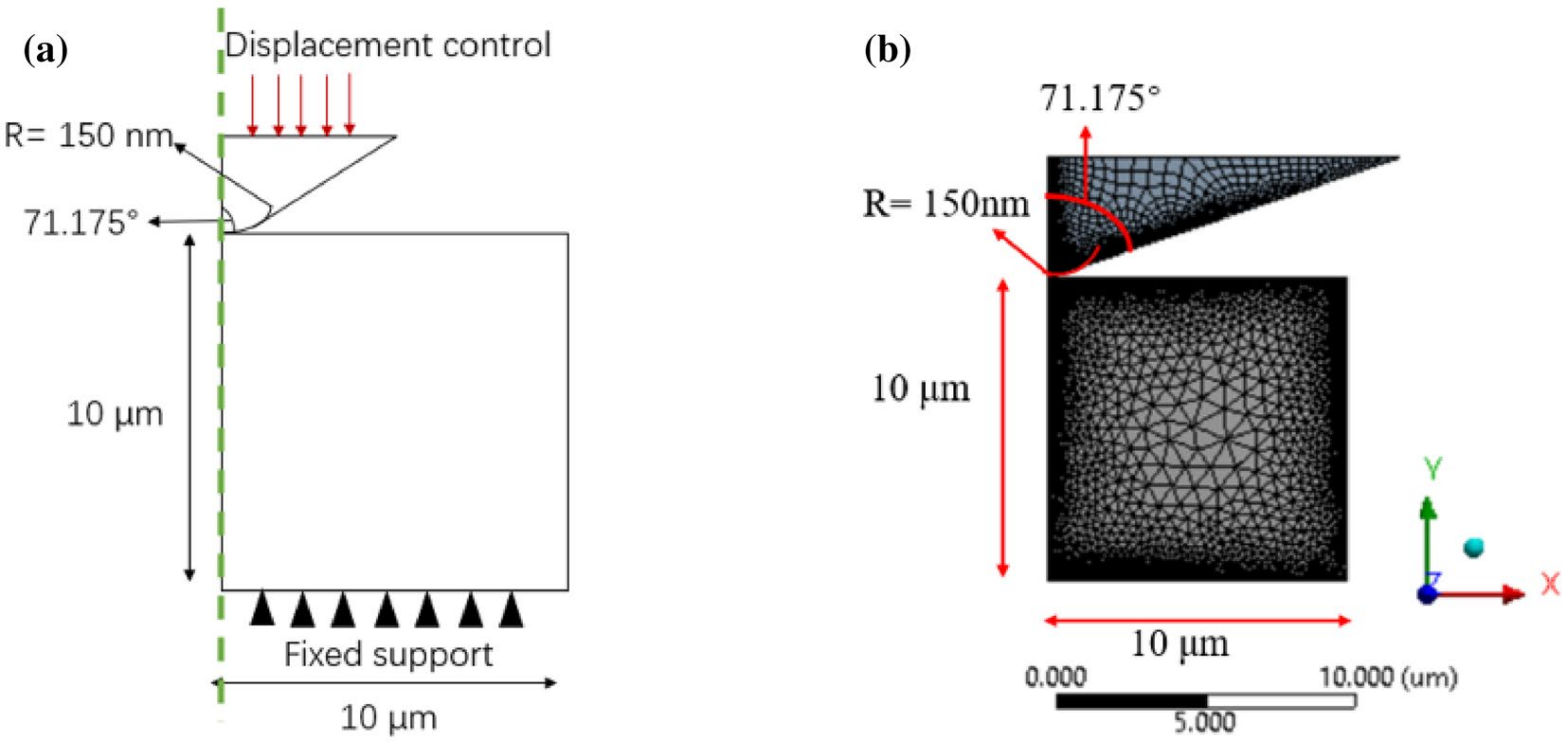
(**a**) 2-D axisymmetric indentation model illustrate a round tip indenter loading on a shale sample which fixed at the bottom. (**b**) Mesh of the specimen with the indenter produced by using Ansys software^26^ (ANSYS-Academic V19.2, https://www.ansys.com/academic/students).

The geometry of the indenter model was based on the nanoindenter that we used experimentally. The probe in this model is drawn based on Berkovich probes. The probe’s angle from one edge to the opposite side is 142.35$^{\circ }$. A typical radius of curvature for a standard Berkovich probe is approximately 150 nm (Fig. 10a).

The indenter is defined by the isotropic elasticity model described by its elastic modulus and Poisson’s ratio. The shale is also defined by the isotropic elasticity model to describe its elastic behavior, in addition, a bilinear kinematic hardening model was used to describe the plasticity behavior of the material. Reduced modulus of shale sample is collected from the nanoindentation experiment. Elastic modulus is calculated by reduced modulus as defined by Eq. (6). Shale’s Poisson’s ratio was obtained from the literature^23^. It should be noted that the temperature dependency of the Poisson’s ratio was ignored in our model. All the parameters used in the numerical model are listed in Table 2.

**Table 2:** The simulation input parameters for the nanoindentation experiment of shale.

	Elastic modulus (GPa)	Poisson’s ratio	Yield stress (MPa)	Tangent modulus (MPa)
Indenter	1140	0.07	NA	NA
Shale	29.815	0.27	600	550

In this numerical study, the displacement control condition was imposed to the top of the indenter. The bottom edge of the model was fixed in all directions and the side edge on the left side was only fixed horizontally. In another word, all the nodes on the axis of symmetry can only move along the axis of symmetry. Since the friction between indenter and sample can be assumed to be negligible, a frictionless contact connection was activated in the setup. With the frictionless connection, the indenter and the sample were separated in the normal direction, and they can also slide against each other in a tangential direction^44^.

Figure 10b illustrates the finite element mesh of the 2-D geometry, quad elements, and triangle elements models that were used for the indenter and shale, respectively. A refine mesh was used near the indenter tip area where the indenter and the sample were in contact. The nodes number on the edges were gradually increased toward the indentation region in order to refine and converge the solution. After convergence, the total node and element numbers were 34657 and 14785, respectively.

## Data Availability

The data will be available upon request.
